# Exploring microRNA target genes and identifying hub genes in bladder cancer based on bioinformatic analysis

**DOI:** 10.1186/s12894-021-00857-w

**Published:** 2021-06-10

**Authors:** Hongjian Wu, Wubing Jiang, Guanghua Ji, Rong Xu, Gaobo Zhou, Hongyuan Yu

**Affiliations:** 1 Department of Urology, Taizhou Hospital of Zhejiang Province, Wenzhou Medical University, Taizhou, 317000 Zhejiang People’s Republic of China; 2 Department of Urology, Taizhou Municipal Hospital, Taizhou, 317000 Zhejiang People’s Republic of China

**Keywords:** Bladder cancer, MiRNAs, MiRNA-mRNA network, Hub genes

## Abstract

**Background:**

Bladder cancer (BC) is the second most frequent malignancy of the urinary system. The aim of this study was to identify key microRNAs (miRNAs) and hub genes associated with BC as well as analyse their targeted relationships.

**Methods:**

According to the microRNA dataset GSE112264 and gene microarray dataset GSE52519, differentially expressed microRNAs (DEMs) and differentially expressed genes (DEGs) were obtained using the R limma software package. The FunRich software database was used to predict the miRNA-targeted genes. The overlapping common genes (OCGs) between miRNA-targeted genes and DEGs were screened to construct the PPI network. Then, gene ontology (GO) analysis was performed through the “cluster Profiler” and “org.Hs.eg.db” R packages. The differential expression analysis and hierarchical clustering of these hub genes were analysed through the GEPIA and UCSC Cancer Genomics Browser databases, respectively. KEGG pathway enrichment analyses of hub genes were performed through gene set enrichment analysis (GSEA).

**Results:**

A total of 12 DEMs and 10 hub genes were identified. Differential expression analysis of the hub genes using the GEPIA database was consistent with the results for the UCSC Cancer Genomics Browser database. The results indicated that these hub genes were oncogenes, but VCL, TPM2, and TPM1 were tumour suppressor genes. The GSEA also showed that hub genes were most enriched in those pathways that were closely associated with tumour proliferation and apoptosis.

**Conclusions:**

In this study, we built a miRNA-mRNA regulatory targeted network, which explores an understanding of the pathogenesis of cancer development and provides key evidence for novel targeted treatments for BC.

## Background

Bladder cancer (BC) is a common urogenital malignancy and is associated with a high recurrence rate and high mortality [1]. Despite great progress in the treatment of BC, its recurrence rate is still high, and its prognosis is poor. Drug resistance and tumour metastasis often contribute to the poor prognosis of bladder cancer. Numerous studies have shown that miRNAs play an important role in these processes. It has been reported that miR-22-3p could enhance chemoresistance by targeting NET1. miRNA-124-3p suppressed cell migration and invasion by targeting ITGA3 signalling in bladder cancer [2], and the miR-626/EYA4 axis was associated with cancer proliferation and metastasis [3]. Accumulating evidence suggests that various miRNAs contribute to cancer initiation, development, and survival prognosis and have potential applications in cancer therapy in numerous malignancies, including BC [4–6].

MicroRNAs (miRNAs) are a series of small endogenous single-stranded noncoding RNAs that are usually approximately 21–25 nucleotides in length. They regulate gene expression post-transcriptionally by binding to the 3’-untranslated region of target mRNAs [7]. Many studies have shown that miRNAs regulate tumour proliferation and apoptosis mainly by regulating mRNA expression [8–10]. miR-133 was reported to play an essential role in muscle development by regulating many genes and has been identified as an important factor in cancer development [11]. miRNA-217 could accelerate the proliferation and migration of BC by inhibiting KMT2D [12]. Thus, there is a regulatory network between miRNAs and mRNAs that can regulate the occurrence, differentiation and development of tumours.

In this study, we screened differentially expressed microRNAs (DEMs) and differentially expressed genes (DEGs) between cancer tissues and normal tissues via bioinformatic analyses. The overlapping common genes (OCGs) between DEGs and DEM-targeted genes were extracted, classified, and extensively analysed. We constructed a miRNA-targeted gene network and investigated a protein–protein interaction (PPI) network along with its hub genes and significant module. Finally, we determined which hub genes and miRNAs might promote the progression of BC, which might help researchers examine molecular mechanisms involved in disease diagnosis, pathogenesis, and prognosis, thus providing information on precise gene therapy for BC research.

## Methods

### Data acquisition and processing

**Fig. 1:**
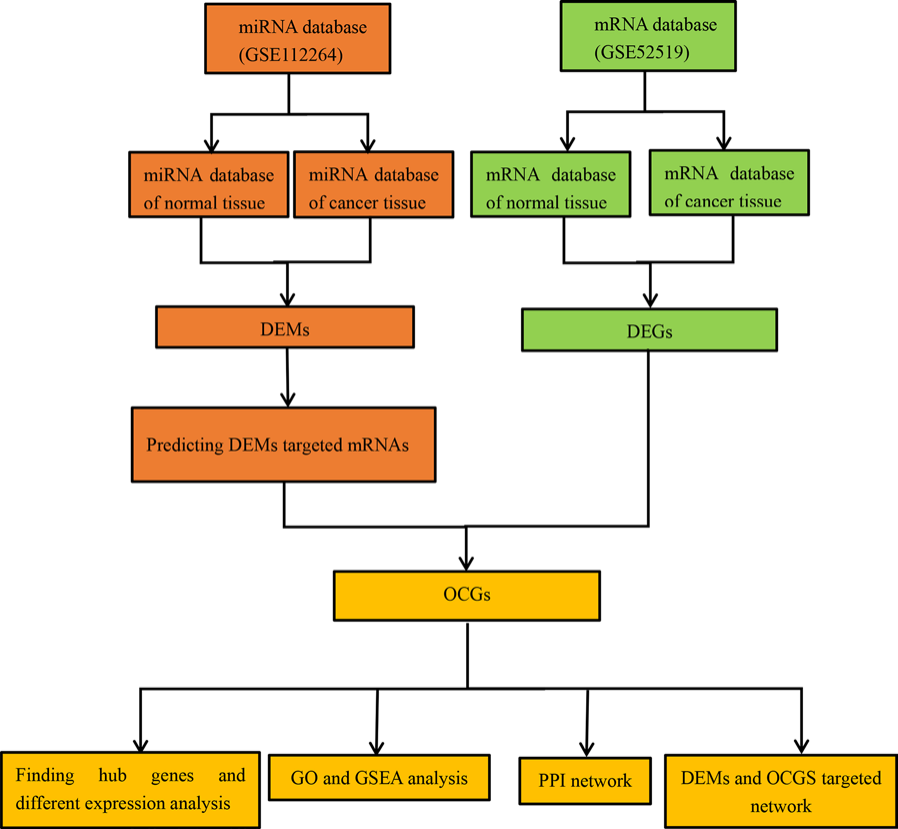
Flowchart of the bioinformatic analysis

We downloaded the gene expression profiles GSE112264 (miRNA) and GSE52519 (mRNA) from the National Center for Biotechnology Information (NCBI) GEO database (https://www.ncbi.nlm.nih.gov/geo). A flowchart was created to illustrate our study programme (Fig. 1). The dataset GSE112264 series contained 50 BC samples and 41 normal controls. The GSE52519 series included 9 cancer tissues and 3 normal controls.

### Identification of DEMs and DEGs

The DEMs and DEGs were obtained from the GEO database using the R language 3.6.1 version limma package. |log fold change (FC)|> 1 and *p*-value < 0.05 were considered statistically significant for the exploration of DEGs and DEMs.

### PPI network construction and analysis

We predicted the miRNA-targeted genes from the FunRich software database [13, 14] and identified OCGs between miRNA-targeted genes and DEGs. The PPI network was built according to these OCGs using the STRING database (http://string-db.org). Finally, we used Cytoscape 3.7.2 [15] and its plug-in cytoHubba to visualize the PPI network.

### GO enrichment analyses of the OCGs

We predicted the potential biological functions of those network genes by Gene Ontology (GO) analysis through the “cluster Profiler” and “org.Hs.eg.db” R packages. A *p*-value < 0.05 was considered statistically significant.

### Hub gene screening and analysis

We used Cytoscape 3.7.2 and its plug-in cytoHubba to find hub genes. Hierarchical clustering of hub genes was built using the UCSC Cancer Genomics Browser (http://genome-cancer.ucsc.edu). The differential expression analysis of hub genes was performed via the GEPIA database (http://gepia.cancer-pku.cn/). To further investigate the potential functions of hub genes, we performed gene set enrichment analysis (GSEA) and multiple GSEA.

### Statistical analysis

All statistical analyses were performed using R version 3.6.1, and a *p*-value < 0.05 was accepted for statistical significance.

## Results

### Identification of DEMs and DEGs

**Fig. 2:**
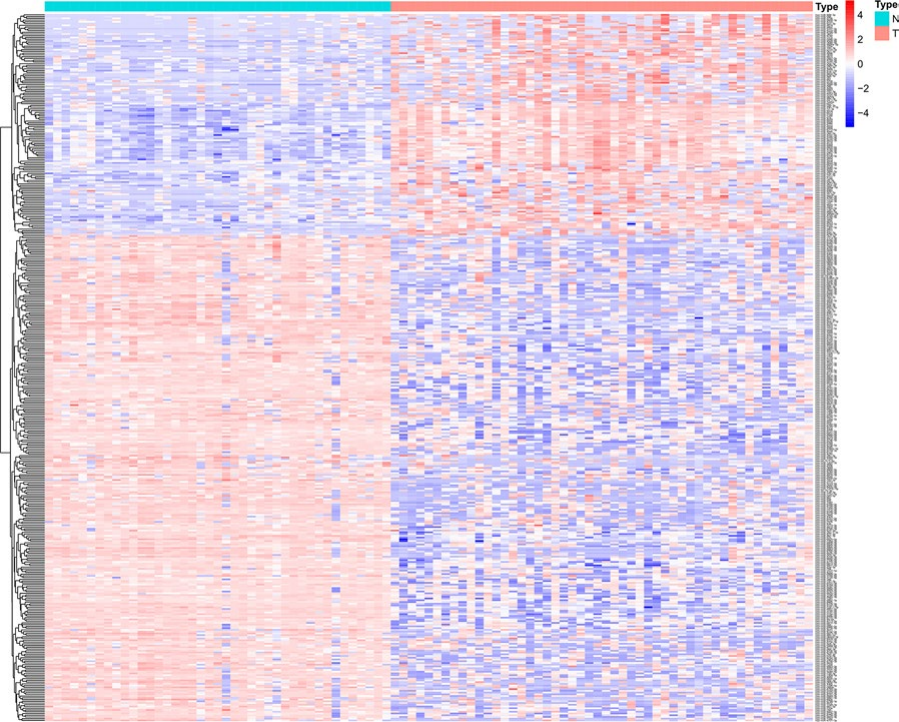
Heat map analysis of the miRNAs between 41 normal samples and 50 tumour samples. Red and blue colours indicate higher expression and lower expression, respectively

Heat map analyses were performed to identify DEGs and DEMs. There were 406 differentially expressed miRNAs, including 127 upregulated and 279 downregulated miRNAs, from the microRNA dataset GSE112264 (Fig. 2). In addition, 370 upregulated genes and 253 downregulated genes were screened from the gene microarray dataset GSE52519.

### Overlapping common gene analysis between DEM-targeted genes and DEGs

**Fig. 3:**
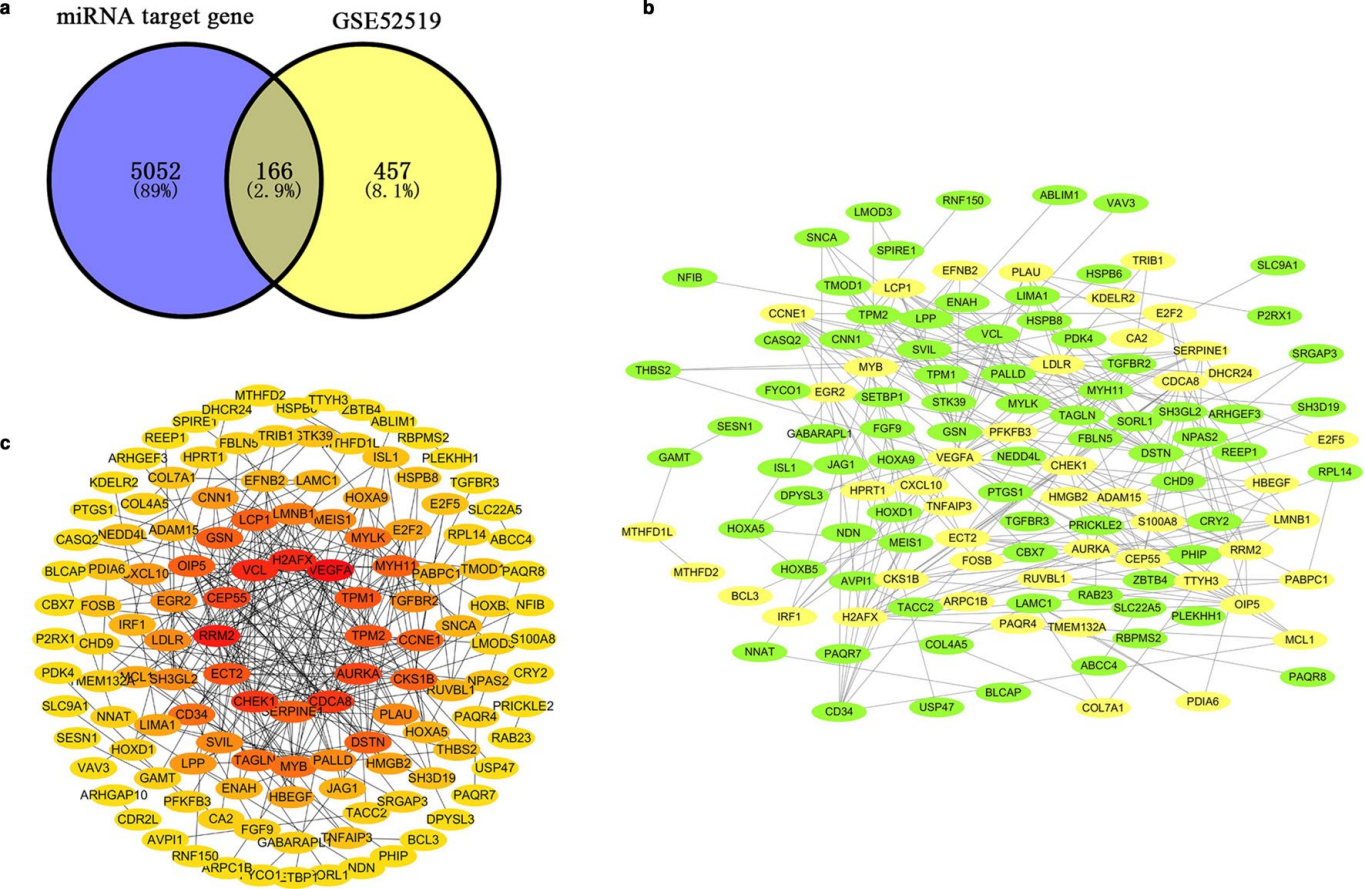
**a** There are 166 OCGs between DEM-targeted genes and DEGs. **b** The PPI network of OCGs was constructed using Cytoscape. Upregulated genes are marked in yellow; downregulated genes are marked in green; **c** The node degrees of OCGs were calculated and visualized using the cytoHubba plug-in of Cytoscape. The redder the colour, the higher the node degree

We screened 5218 DEM-targeted genes according to the FunRich database. After determining the overlap between DEM-targeted genes and DEGs through a Venn diagram, we found 166 OCGs (Fig. 3a) and used the STRING database to visualize the protein relationship. Then, we built a protein–protein interaction (PPI) network (Fig. 3b) and calculated the protein node degrees through cytoHubba (Fig. 3c).

### GO enrichment analyses of OCGs

**Fig. 4:**
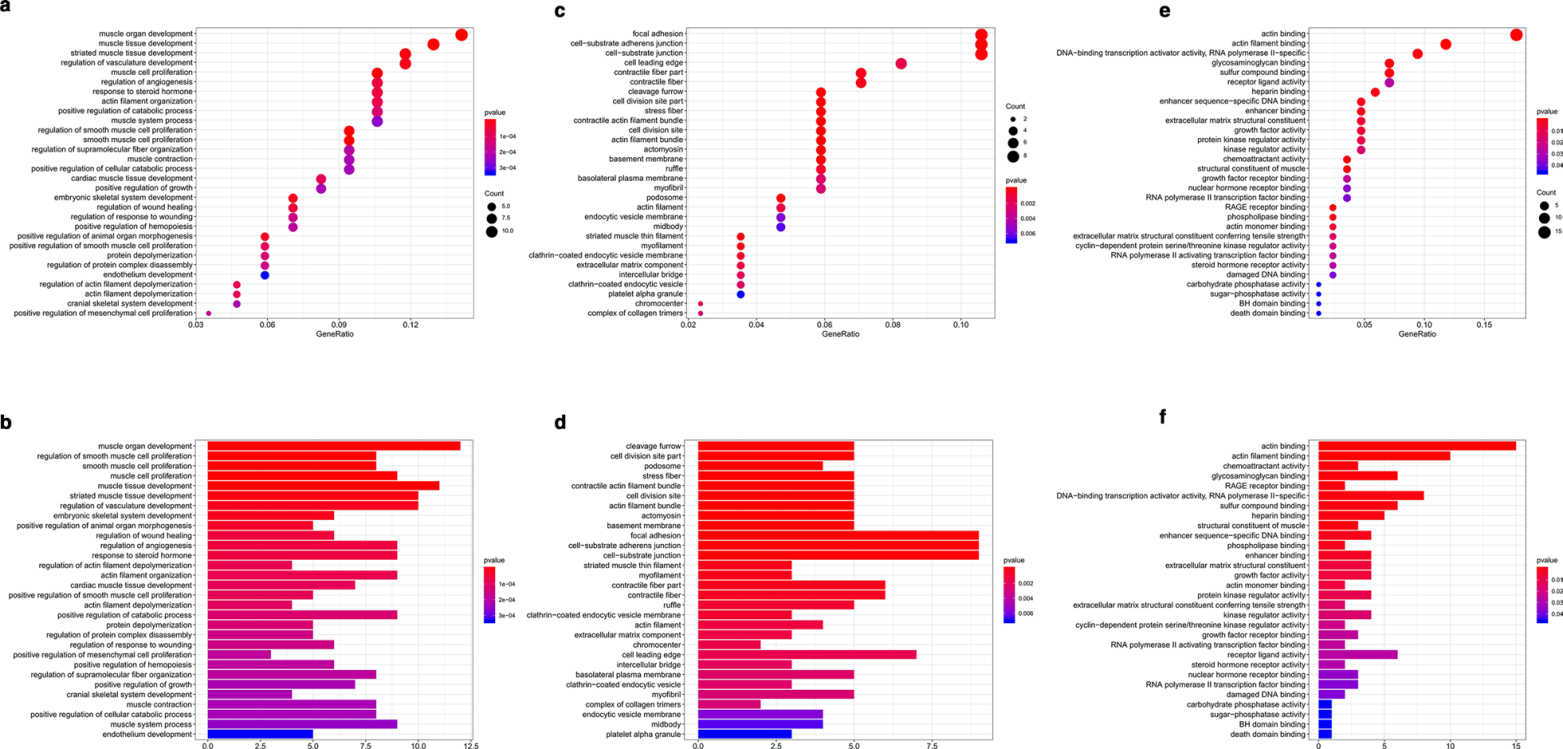
GO biological function enrichment analysis of the OCGs; **a**, **b** Biological process (BP); **c**, **d** Cellular component (CC); **e**, **f** Molecular function (MF)

We predicted the potential biological functions of these genes through GO enrichment analysis. GO analysis results were listed in Table 1. The top ten terms of GO annotation in biological process (BP), cellular component (CC) and molecular function (MF) were displayed. The BP-enriched functions were mainly associated with muscle organ development and muscle cell proliferation (Fig. 4a, b). The CC biological functions were mainly enriched in focal adhesion and cell substrate adherent junctions (Fig. 4c, d). The MF biological functions were mainly enriched in actin binding and actin filament binding (Fig. 4e, f).

**Table 1:** GO enrichment analysis

Term	ID	Description	Count	*p*-value
BP	GO:0007517	Muscle organ development	12	3.32E−07
BP	GO:0060537	Muscle tissue development	11	2.37E−06
BP	GO:0014706	Striated muscle tissue development	10	1.09E−05
BP	GO:1901342	Regulation of vasculature development	10	2.16E−05
BP	GO:0033002	Muscle cell proliferation	9	1.40E−06
BP	GO:0045765	Regulation of angiogenesis	9	6.10E−05
BP	GO:0048545	Response to steroid hormone	9	6.35E−05
BP	GO:0007015	Actin filament organization	9	8.51E−05
BP	GO:0009896	Positive regulation of catabolic process	9	1.30E−04
BP	GO:0003012	Muscle system process	9	2.63E−04
CC	GO:0005925	Focal adhesion	9	6.18E−05
CC	GO:0005924	Cell-substrate adherens junction	9	6.54E−05
CC	GO:0030055	Cell-substrate junction	9	7.05E−05
CC	GO:0031252	Cell leading edge	7	0.001776937
CC	GO:0044449	Contractile fiber part	6	3.88E−04
CC	GO:0043292	Contractile fiber	6	5.25E−04
CC	GO:0062023	Collagen-containing extracellular matrix	6	0.008196759
CC	GO:0032154	Cleavage furrow	5	3.88E−06
CC	GO:0032155	Cell division site part	5	7.63E−06
CC	GO:0001725	Stress fiber	5	1.03E−05
MF	GO:0003779	Actin binding	15	1.87E−09
MF	GO:0051015	Actin filament binding	10	3.70E−08
MF	GO:0001228	DNA-binding transcription activator activity, RNA polymerase ii-specific	8	0.00122079
MF	GO:0005539	Glycosaminoglycan binding	6	8.21E−04
MF	GO:1901681	Sulfur compound binding	6	0.001290131
MF	GO:0048018	Receptor ligand activity	6	0.028441857
MF	GO:0008201	Heparin binding	5	0.00132645
MF	GO:0001158	Enhancer sequence-specific DNA binding	4	0.002587729
MF	GO:0035326	Enhancer binding	4	0.003859145
MF	GO:0005201	Extracellular matrix structural constituent	4	0.007879546

### The network between DEMs and hub genes

**Fig. 5:**
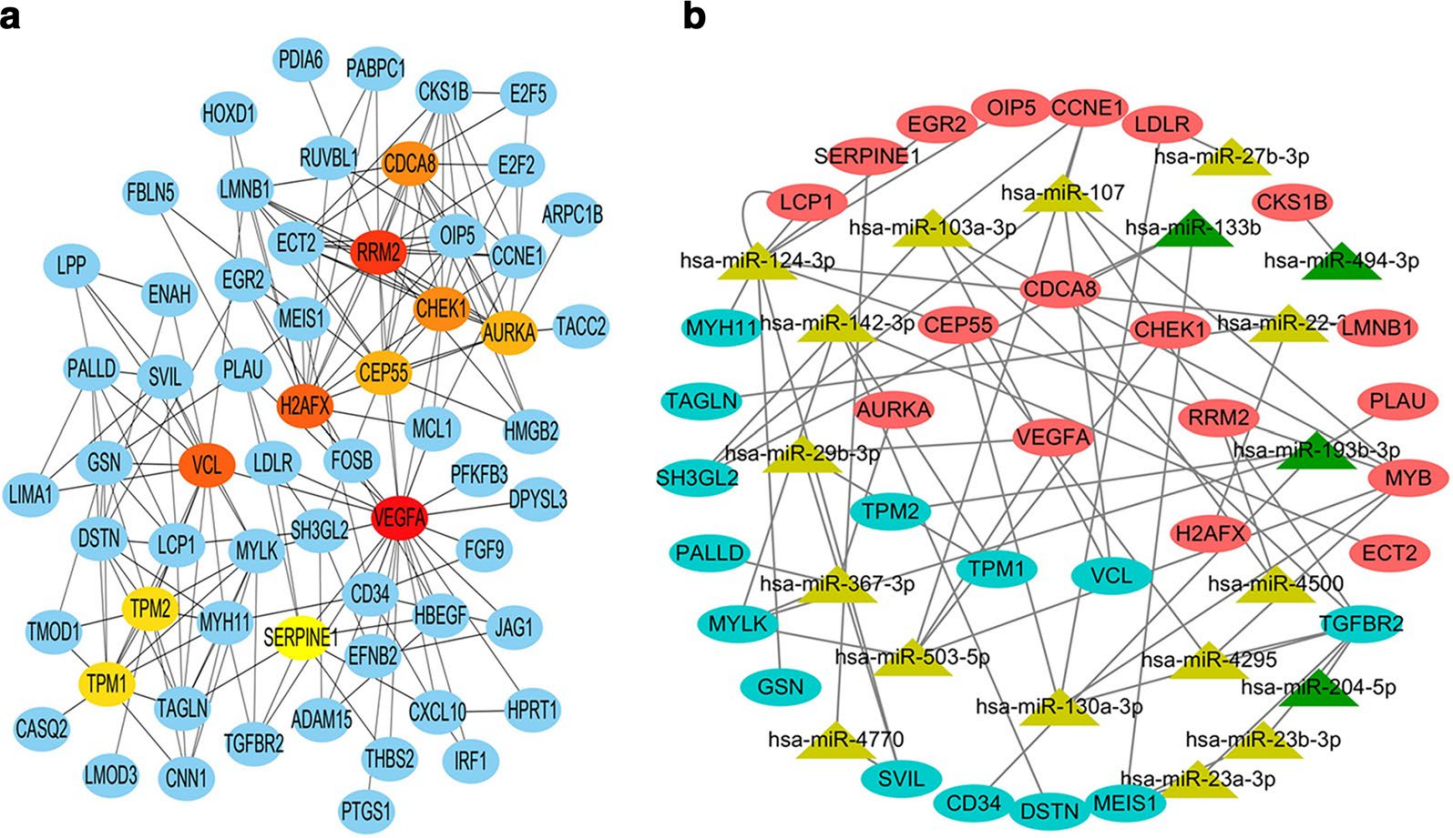
**a** Ten hub genes according to node degrees using the cytoHubba plug-in of Cytoscape. **b** The network between DEMs and hub genes was visualized using Cytoscape software. Upregulated genes are marked in red; downregulated genes are marked in blue; Upregulated miRNAs are marked in yellow; downregulated miRNAs are marked in green

Ten hub genes were identified according to the node degrees (Table 2) through Cytoscape and its plug-in cytoHubba (Fig. 5a). Then, the network between DEMs and hub genes was visualized through Cytoscape software (Fig. 5b). According to the miRNA-mRNA targeted network, the network included hsa-miR-103a-3p, hsa-miR-107, hsa-miR-130a-3p, hsa-miR-133b, hsa-miR-142-3p, hsa-miR-193b-3p, hsa-miR-22-3p, hsa-miR-29b-3p, hsa-miR-367-3p, hsa-miR-4295, hsa-miR-4500 and hsa-miR-503-5p, which had targeted relationships with the hub genes. The top 10 hub genes included vascular endothelial growth factor A (VEGFA), ribonucleotide reductase regulatory subunit M2 (RRM2), H2A histone family member X (H2AFX), vinculin (VCL), cell division cycle associated 8 (CDCA8), checkpoint kinase 1 (CHEK1), aurora kinase A (AURKA), centrosomal protein 55 (CEP55), tropomyosin 2 (TPM2), and tropomyosin 1 (TPM1), and their target miRNAs are shown in Table 3.

**Table 2:** Hub genes in the PPI network

Gene name	Node degree	Closeness	Clustering coefficient	EPC	Radiality	Stress
VEGFA	24.000	58.700	0.091	40.625	6.663	13,708.000
RRM2	16.000	51.350	0.392	39.954	6.387	5036.000
VCL	14.000	49.817	0.308	38.092	6.348	6068.000
H2AFX	14.000	48.833	0.264	38.408	6.269	4058.000
CHEK1	13.000	48.683	0.577	39.721	6.301	2348.000
CDCA8	13.000	44.733	0.564	39.174	6.025	1232.000
CEP55	12.000	47.767	0.470	38.197	6.269	2870.000
AURKA	12.000	44.067	0.545	37.567	6.009	2206.000
TPM1	11.000	42.700	0.491	36.769	5.938	1214.000
TPM2	11.000	42.867	0.491	36.542	5.946	1838.000

**Table 3:** The relation between miRNAs and hub genes

miRNA	Gene	miRNA logFC	Gene logFC	Regulate
hsa-miR-29b-3p	VEGFA	4.57576096	1.188669972	Up
hsa-miR-503-5p	VEGFA	2.1294029	1.188669972	Up
hsa-miR-103a-3p	VCL	2.832553104	− 1.172441766	Down
hsa-miR-107	VCL	2.203911303	− 1.172441766	Down
hsa-miR-193b-3p	TPM2	− 2.053746722	− 3.040805396	Down
hsa-miR-29b-3p	TPM1	4.57576096	− 2.285811133	Down
hsa-miR-142-3p	TPM1	2.067522814	− 2.285811133	Down
hsa-miR-4500	RRM2	2.013295449	1.193739332	Up
hsa-miR-22-3p	H2AFX	2.663671233	1.144533464	Up
hsa-miR-503-5p	CHEK1	2.1294029	1.302980041	Up
hsa-miR-130a-3p	CEP55	2.394392517	1.408940672	Up
hsa-miR-4295	CEP55	2.456498239	1.408940672	Up
hsa-miR-4500	CDCA8	2.013295449	1.353842603	Up
hsa-miR-133b	CDCA8	− 2.383502249	1.353842603	Up
hsa-miR-367-3p	AURKA	2.011552364	1.804765390	Up

*FC* Fold change

### Hub gene expression and functional analysis

**Fig. 6:**
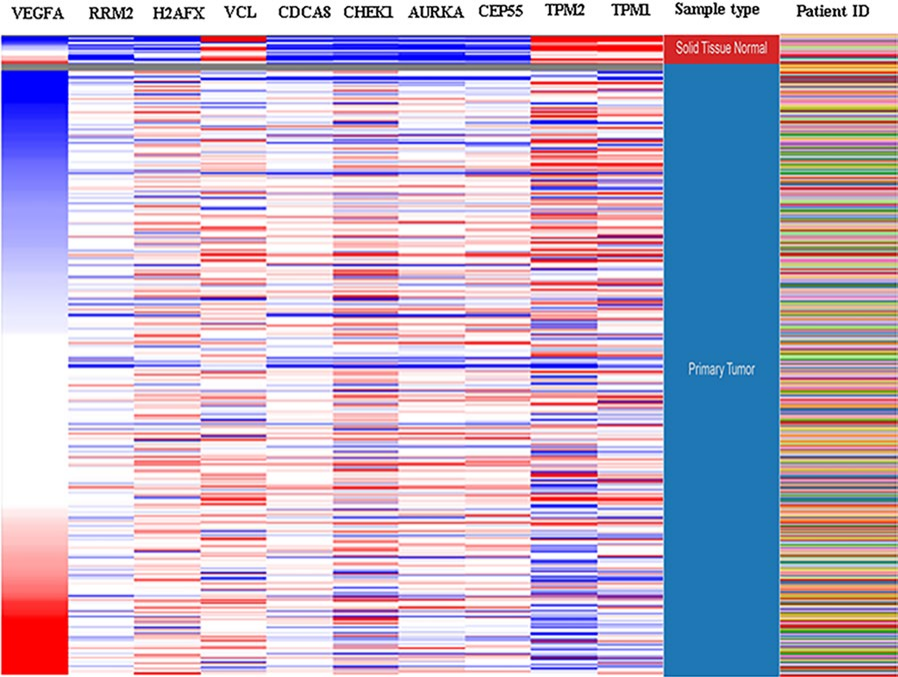
Hierarchical clustering of hub genes was performed using the UCSC database. The samples under the red bar are normal samples, and the samples under the blue bar are BC samples. The upregulation of genes is marked in red and downregulation of genes is marked in blue

**Fig. 7:**
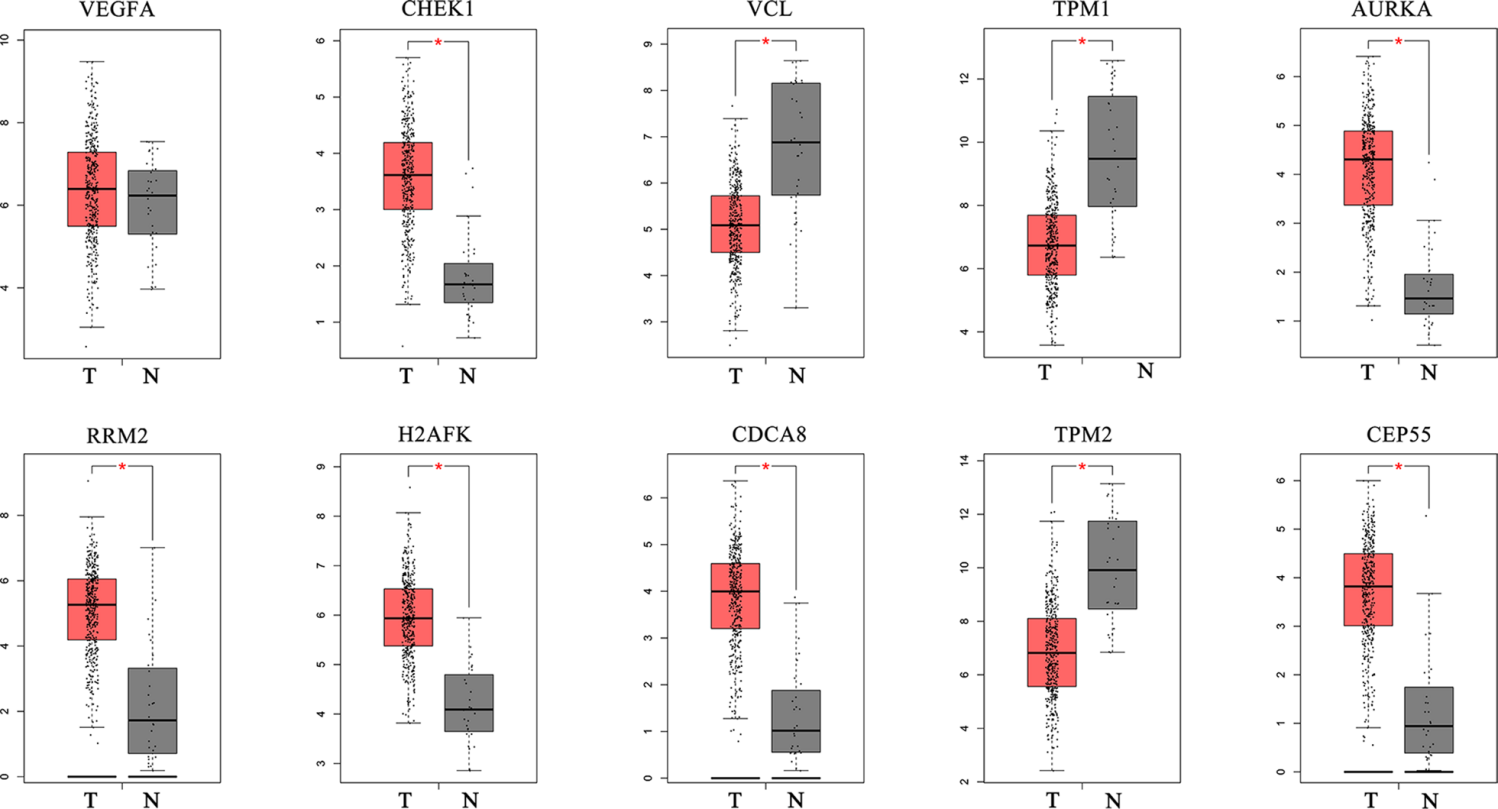
Expression analysis of normal (N) versus tumour (T) tissue through the GEPIA database. The asterisk is considered statistically significant (*p* < 0.05)

**Fig. 8:**
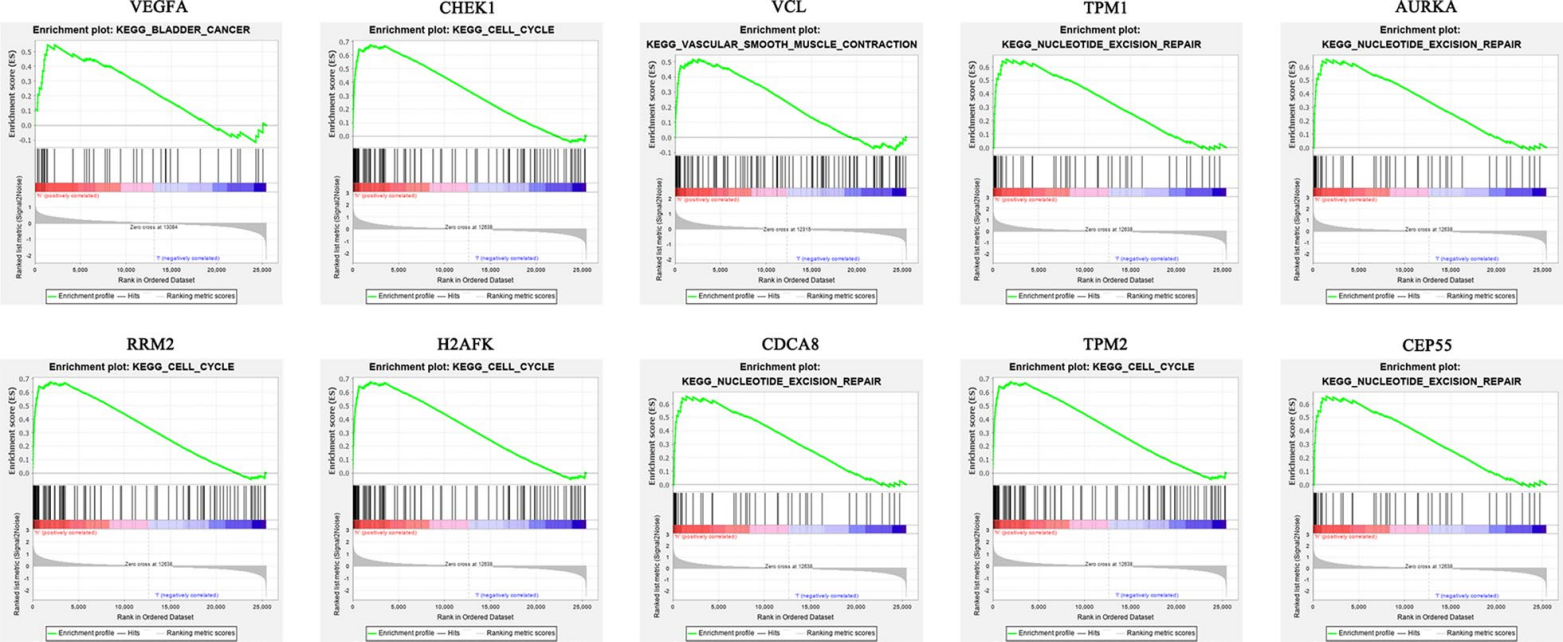
GSEA of hub genes

**Fig. 9:**
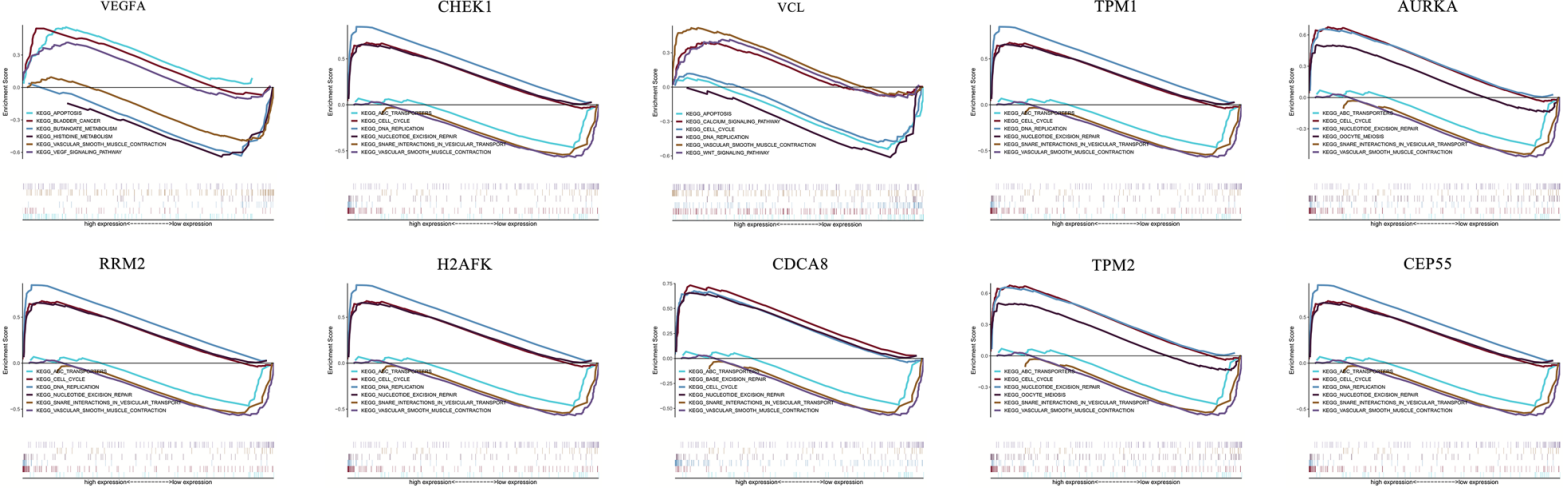
Multiple GSEA of hub genes

Hierarchical clustering analysis indicated that the hub genes were significantly differentially expressed between the normal and tumour tissues (Fig. 6). GEPIA database analysis indicated that those hub genes were significantly upregulated in the tumour tissues; however, VCL, TPM2, and TPM1 were significantly downregulated (Fig. 7). This result was consistent with hierarchical clustering analysis. Therefore, the results indicated that these hub genes were oncogenes, but VCL, TPM2, and TPM1 were tumour suppressor genes. To further investigate the potential functions of the hub genes, we performed GSEA. The high enrichment plot of those hub genes was most enriched in the “cell cycle” and “nucleotide excision repair” pathways. VEGFA was most enriched in bladder cancer, and VCL was highly related to vascular smooth muscle contraction (Fig. 8). Multiple GSEA also showed that these hub genes with lower expression levels were most enriched in the “vascular smooth muscle contraction”, “snare interactions in vesicular transport” and “abc transporters” pathways. The hub genes with higher expression levels were most enriched in the “cell cycle”, “nucleotide excision repair” and “cell apoptosis” pathways (Fig. 9). These pathways were closely associated with tumour proliferation and apoptosis.

## Discussion

miRNAs regulate the progression of tumours by regulating their target genes, and some miRNAs have been identified as being involved in several types of cancer [16, 17]. Therefore, it is of great significance to study relationships between miRNAs and targeted genes in BC. In our study, we screened the DEGs and DEMs through GSE52519 and GSE112264 expression profiles, respectively. There were 127 upregulated and 279 downregulated miRNAs that were differentially expressed in BC patients. We found that hsa-miR-103a-3p, hsa-miR-107, hsa-miR-130a-3p, hsa-miR-133b, hsa-miR-142-3p, hsa-miR-193b-3p, hsa-miR-22-3p, hsa-miR-29b-3p, hsa-miR-367-3p, hsa-miR-4295, hsa-miR-4500 and hsa-miR-503-5p were potential core miRNAs that play an important role in the development and prognosis of BC.

hsa-miR-29b-3p was the most significantly expressed in the tumour tissues and regulated two hub genes, VEGFA and TPM1. hsa-miR-29b-3p plays an important role in many pathophysiological processes, such as cell proliferation, apoptosis and metastasis [18–20], and these processes have been reported in a series of tumour types [21–23]. hsa-miR-29b-3p could control tumour proliferation, invasion and angiogenesis by regulating VEGFA [24]. Chou et al. [25] reported that the hsa-miR‐29b-VEGFA axis was closely related to metastasis and deterioration in breast cancer. Szczyrba et al. [23] showed that induced VEGF protein expression regulated by hsa-miR‐29b was involved in cancer development. It was shown that hsa-miR-103a-3p played an important role in bladder functions and was significantly correlated with patient survival [26, 27]. Moreover, some studies showed that miR-103a-3p and hsa-miR-107 were significantly upregulated in tumour tissues of BC[28], and they were positively associated with tumour stages [29]. hsa-miR-133b was also associated with the prognosis of patients [30], and hsa-miR-142-3p was upregulated in bladder urothelial carcinoma patients [31]. miR-193b-3p could downregulate the cell cycle to inhibit cell proliferation [32]. Promoting miR-4295 transcription could reduce p63α translation and enhance urothelial transformation for tumorigenesis [33]. As a result, these miRNAs have been reported to be closely correlated with the occurrence and development of BC.

We selected 10 hub genes according to node degrees, such as VEGFA, VCL, TPM2, TPM1, RRM2, H2AFX, CHEK1, CEP55, CDCA8 and AURKA. In the present study, VEGFA, RRM2, H2AFX, CHEK1, CEP55, CDCA8 and AURKA were significantly upregulated; however, VCL, TPM2 and TPM1 were significantly downregulated in cancer samples of BC. Furthermore, GEPIA database analysis showed that the result was consistent with the hierarchical clustering analysis and indicated that VCL, TPM2 and TPM1 were tumour suppressor genes and the others were oncogenic genes. Our study showed that VEGFA was the core of these hub genes, which further validated its important role in tumour deterioration and development. VEGFA is a member of the VEGF growth factor family, which can promote the proliferation and migration of vascular endothelial cells. VEGFA is significantly upregulated in many cancers and is essential for tumour proliferation, invasion and metastasis [34, 35]. VCL is a cytoskeletal protein associated with cell–cell and cell–matrix junctions, where it is thought to function as one of several interacting proteins involved in anchoring F-actin to the membrane. Recent research has shown that morphological and mechanical stability are directly related to the actin filament organization used by cancer cells to adapt to altered laminin-rich microenvironments [36]. VCL might affect cancer development through the focal adhesion pathway [37]. TPM2 encodes beta-tropomyosin, a member of the actin filament binding protein family that is poorly expressed in high-grade, relapsed, and metastatic prostate tumours and may be a potential prognostic biomarker in prostate cancer [38]. TPM1 is also a member of the tropomyosin family of highly conserved, widely distributed actin-binding proteins involved in the contractile system of striated and smooth muscles and the cytoskeleton of non-muscle cells. The upregulation of TPM1 inhibited cell proliferation, induced apoptosis, and inhibited cancer growth in BC cells [39]. Moreover, the migratory and invasion ability of cancer cells was enhanced by the destruction of stress fibres and by TPM1-mediated relevant adhesive structures [40, 41]. Additionally, Thorsen et al. [42] also revealed that the mRNA expression level and protein expression level of TPM1 were downregulated in BC. These results supported our idea of TPM1 as a tumour suppressor. To further investigate the potential functions of these hub genes, we performed GSEA and multiple GSEA. The results showed that these hub genes were most enriched in those pathways that were closely associated with tumour proliferation and apoptosis.

## Conclusions

In conclusion, we constructed a miRNA-mRNA regulatory network and showed the connections between hub genes and miRNAs in the regulatory network. This study indicated the potential mechanisms of the miRNA-mRNA regulatory network in the physiological and pathological processes of BC, and it might provide insight into therapy and improve prognosis in the future. Since the number of gene chips included in this study is not large, there may be some shortcomings and contradictions, such as data bias and differences in detection methods. Thus, we are planning to further perform a series of experiments to verify the results of this study and explore the molecular mechanism of the regulatory network.

## Data Availability

Publicly available datasets were analysed in this study. This data can be found here: Gene Expression Omnibus (GEO) database (https://www.ncbi.nlm.nih.gov/geo).

## Abbreviations

BC: Bladder cancer
miRNAs: MicroRNAs
DEMs: Differentially expressed microRNAs
DEGs: Differentially expressed genes
OCGs: Overlapping common genes
PPI: Protein–protein interaction
KEGG: Kyoto Encyclopedia of Genes and Genomes
GO: Gene Ontology
BP: Biological process
CC: Cellular component
MF: Molecular function
GSEA: Gene Set Enrichment Analysis
VEGFA: Vascular endothelial growth factor A
RRM2: Ribonucleotide reductase regulatory subunit M2
H2AFX: H2A histone family member X
VCL: Vinculin
CDCA8: Cell division cycle associated 8
CHEK1: Checkpoint kinase 1
AURKA: Aurora kinase A
CEP55: Centrosomal protein 55
TPM2: Tropomyosin 2
TPM1: Tropomyosin 1
GEO: Gene expression omnibus

## References

[1] Martinez Rodriguez RH, Buisan Rueda O, Ibarz L (2017). Bladder cancer: present and future. Med Clin (Barc).

[2] Wang JR, Liu B, Zhou L, Huang YX (2019). MicroRNA-124-3p suppresses cell migration and invasion by targeting ITGA3 signaling in bladder cancer. Cancer Biomark.

[3] Dong W, Bi JM, Liu HW, Yan D, He QQ, Zhou QH, Wang Q, Xie RH, Su YJ, Yang MH (2019). Circular RNA ACVR2A suppresses bladder cancer cells proliferation and metastasis through miR-626/EYA4 axis. Mol Cancer.

[4] Babaei K, Shams S, Keymoradzadeh A, Vahidi S, Hamami P, Khaksar R, Norollahi SE, Samadani AA (2020). An insight of microRNAs performance in carcinogenesis and tumorigenesis; an overview of cancer therapy. Life Sci.

[5] Calin GA, Croce CM (2006). MicroRNA signatures in human cancers. Nat Rev Cancer.

[6] Enokida H, Yoshino H, Matsushita R, Nakagawa M (2016). The role of microRNAs in bladder cancer. Investig Clin Urol.

[7] Bartel DP (2009). MicroRNAs: target recognition and regulatory functions. Cell.

[8] Amuran GG, Eyuboglu IP, Tinay I, Akkiprik M (2018). New Insights in Bladder Cancer Diagnosis: Urinary miRNAs and Proteins. Med Sci (Basel).

[9] Dong F, Xu T, Shen Y, Zhong S, Chen S, Ding Q, Shen Z (2017). Dysregulation of miRNAs in bladder cancer: altered expression with aberrant biogenesis procedure. Oncotarget.

[10] Kutwin P, Konecki T, Borkowska EM, Traczyk-Borszynska M, Jablonowski Z (2018). Urine miRNA as a potential biomarker for bladder cancer detection: a meta-analysis. Cent European J Urol.

[11] Yu H, Lu Y, Li Z, Wang Q (2014). microRNA-133: expression, function and therapeutic potential in muscle diseases and cancer. Curr Drug Targets.

[12] Hou G, Xu W, Jin Y, Wu J, Pan Y, Zhou F (2019). MiRNA-217 accelerates the proliferation and migration of bladder cancer via inhibiting KMT2D. Biochem Biophys Res Commun.

[13] Pathan M, Keerthikumar S, Chisanga D, Alessandro R, Ang CS, Askenase P, Batagov AO, Benito-Martin A, Camussi G, Clayton A (2017). A novel community driven software for functional enrichment analysis of extracellular vesicles data. J Extracell Vesicles.

[14] Benito-Martin A, Peinado H (2015). FunRich proteomics software analysis, let the fun begin!. Proteomics.

[15] Shannon P, Markiel A, Ozier O, Baliga NS, Wang JT, Ramage D, Amin N, Schwikowski B, Ideker T (2003). Cytoscape: a software environment for integrated models of biomolecular interaction networks. Genome Res.

[16] Ying Y, Li J, Xie H, Yan H, Jin K, He L, Ma X, Wu J, Xu X, Fang J (2020). CCND1, NOP14 and DNMT3B are involved in miR-502-5p-mediated inhibition of cell migration and proliferation in bladder cancer. Cell Prolif.

[17] Wang F, Wu H, Fan M, Yu R, Zhang Y, Liu J, Zhou X, Cai Y, Huang S, Hu Z (2020). Sodium butyrate inhibits migration and induces AMPK-mTOR pathway-dependent autophagy and ROS-mediated apoptosis via the miR-139-5p/Bmi-1 axis in human bladder cancer cells. FASEB J.

[18] Li Y, Wang F, Xu J, Ye F, Shen Y, Zhou J, Lu W, Wan X, Ma D, Xie X (2011). Progressive miRNA expression profiles in cervical carcinogenesis and identification of HPV-related target genes for miR-29. J Pathol.

[19] Martinez I, Cazalla D, Almstead LL, Steitz JA, DiMaio D (2011). miR-29 and miR-30 regulate B-Myb expression during cellular senescence. Proc Natl Acad Sci U S A.

[20] Ugalde AP, Ramsay AJ, de la Rosa J, Varela I, Marino G, Cadinanos J, Lu J, Freije JM, Lopez-Otin C (2011). Aging and chronic DNA damage response activate a regulatory pathway involving miR-29 and p53. EMBO J.

[21] Espinosa-Parrilla Y, Munoz X, Bonet C, Garcia N, Vencesla A, Yiannakouris N, Naccarati A, Sieri S, Panico S, Huerta JM (2014). Genetic association of gastric cancer with miRNA clusters including the cancer-related genes MIR29, MIR25, MIR93 and MIR106: results from the EPIC-EURGAST study. Int J Cancer.

[22] Sandhu R, Rivenbark AG, Mackler RM, Livasy CA, Coleman WB (2014). Dysregulation of microRNA expression drives aberrant DNA hypermethylation in basal-like breast cancer. Int J Oncol.

[23] Szczyrba J, Nolte E, Hart M, Doll C, Wach S, Taubert H, Keck B, Kremmer E, Stohr R, Hartmann A (2013). Identification of ZNF217, hnRNP-K, VEGF-A and IPO7 as targets for microRNAs that are downregulated in prostate carcinoma. Int J Cancer.

[24] Zhao X, Liu Y, Li Z, Zheng S, Wang Z, Li W, Bi Z, Li L, Jiang Y, Luo Y (2018). Linc00511 acts as a competing endogenous RNA to regulate VEGFA expression through sponging hsa-miR-29b-3p in pancreatic ductal adenocarcinoma. J Cell Mol Med.

[25] Chou J, Lin JH, Brenot A, Kim JW, Provot S, Werb Z (2013). GATA3 suppresses metastasis and modulates the tumour microenvironment by regulating microRNA-29b expression. Nat Cell Biol.

[26] Jiang X, Du L, Duan W, Wang R, Yan K, Wang L, Li J, Zheng G, Zhang X, Yang Y (2016). Serum microRNA expression signatures as novel noninvasive biomarkers for prediction and prognosis of muscle-invasive bladder cancer. Oncotarget.

[27] Gheinani AH, Kiss B, Moltzahn F, Keller I, Bruggmann R, Rehrauer H, Fournier CA, Burkhard FC, Monastyrskaya K (2017). Characterization of miRNA-regulated networks, hubs of signaling, and biomarkers in obstruction-induced bladder dysfunction. JCI Insight.

[28] Zhong Z, Lv M, Chen J (2016). Screening differential circular RNA expression profiles reveals the regulatory role of circTCF25-miR-103a-3p/miR-107-CDK6 pathway in bladder carcinoma. Sci Rep.

[29] Yu QF, Liu P, Li ZY, Zhang CF, Chen SQ, Li ZH, Zhang GY, Li JC (2018). MiR-103/107 induces tumorigenicity in bladder cancer cell by suppressing PTEN. Eur Rev Med Pharmacol Sci.

[30] Shi Z, Kadeer A, Wang M, Wen B, Li M, Huang J, Gao Y, Liu E, Liu D, Jia D (2019). The deregulation of miR-133b is associated with poor prognosis in bladder cancer. Pathol Res Pract.

[31] Zhang ZC, Huang Y, Wang XJ, Wang M, Ma LL (2013). Expression of circulating microRNAs in patients with bladder urothelial carcinoma. Beijing Da Xue Xue Bao Yi Xue Ban.

[32] Lin SR, Yeh HC, Wang WJ, Ke HL, Lin HH, Hsu WC, Chao SY, Hour TC, Wu WJ, Pu YS (2017). MiR-193b mediates CEBPD-induced cisplatin sensitization through targeting ETS1 and cyclin D1 in human urothelial carcinoma cells. J Cell Biochem.

[33] Jin H, Xu J, Guo X, Huang H, Li J, Peng M, Zhu J, Tian Z, Wu XR, Tang MS (2016). XIAP RING domain mediates miR-4295 expression and subsequently inhibiting p63alpha protein translation and promoting transformation of bladder epithelial cells. Oncotarget.

[34] Paez-Ribes M, Allen E, Hudock J, Takeda T, Okuyama H, Vinals F, Inoue M, Bergers G, Hanahan D, Casanovas O (2009). Antiangiogenic therapy elicits malignant progression of tumors to increased local invasion and distant metastasis. Cancer Cell.

[35] Yu X, Ye F (2020). Role of Angiopoietins in Development of Cancer and Neoplasia Associated with Viral Infection. Cells.

[36] Lekka M, Pabijan J, Orzechowska B (2019). Morphological and mechanical stability of bladder cancer cells in response to substrate rigidity. Biochim Biophys Acta Gen Subj.

[37] Wu Z, Wang S, Jiang F, Li Q, Wang C, Wang H, Zhang W, Xue P, Wang SL (2017). Mass spectrometric detection combined with bioinformatic analysis identified possible protein markers and key pathways associated with bladder cancer. Gene.

[38] Varisli L (2013). Identification of new genes downregulated in prostate cancer and investigation of their effects on prognosis. Genet Test Mol Biomark.

[39] Liu G, Zhao X, Zhou J, Cheng X, Ye Z, Ji Z (2018). Long non-coding RNA MEG3 suppresses the development of bladder urothelial carcinoma by regulating miR-96 and TPM1. Cancer Biol Ther.

[40] Redwood C, Robinson P (2013). Alpha-tropomyosin mutations in inherited cardiomyopathies. J Muscle Res Cell Motil.

[41] Lin J, Shen J, Yue H, Cao Z (2019). miRNA1835p1 promotes the migration and invasion of gastric cancer AGS cells by targeting TPM1. Oncol Rep.

[42] Thorsen K, Sorensen KD, Brems-Eskildsen AS, Modin C, Gaustadnes M, Hein AM, Kruhoffer M, Laurberg S, Borre M, Wang K (2008). Alternative splicing in colon, bladder, and prostate cancer identified by exon array analysis. Mol Cell Proteomics.

